# Evaluating effectiveness of an integrated return-to-work and vocational rehabilitation program on work disability duration in the construction sector

**DOI:** 10.5271/sjweh.4006

**Published:** 2022-03-31

**Authors:** Robert A Macpherson, Ailin He, Benjamin C Amick III, Mieke Koehoorn, Christopher B McLeod

**Affiliations:** 1 Partnership for Work, Health and Safety, School of Population and Public Health, University of British Columbia, Vancouver, Canada; 2 Fay W. Boozman College of Public Health, University of Arkansas for Medical Sciences, Little Rock, Arkansas, United States; 3 Winthrop P. Rockefeller Cancer Institute, University of Arkansas for Medical Sciences, Little Rock, Arkansas, United States; 4 Institute for Work & Health, Toronto, Ontario, Canada

**Keywords:** difference-in-difference, observational study, observational study design, occupational rehabilitation, Ontario, sickness absence, sick leave, quasi-experimental study, work disability absence, work-related injury, work-related musculoskeletal disorder, worker compensation

## Abstract

**Objective:**

The aim of this study was to investigate whether an integrated return-to-work (RTW) and vocational rehabilitation (VR) program – the Work Reintegration (WR) program – was associated with reduced work disability duration in the construction sector in Ontario, Canada.

**Methods:**

Workers’ compensation data from the Ontario Workplace Safety and Insurance Board were extracted for lost-time construction worker claims following work-related injuries between 2009 and 2015. Claims receiving referrals to RTW and VR specialists (treatments) were matched with claims receiving no referrals (controls) during the periods before and after the WR program introduction. Multivariable difference-in-differences linear and quantile regression models were used to examine differences in cumulative disability days paid during two-years post-injury between treatment and control groups before and after the program change and the difference in these differences, overall, and at different disability distribution percentiles.

**Results:**

The WR program introduction was associated with reductions in cumulative disability days paid for all claims but most notably among longer duration claims referred to RTW specialists (reduction of 274 days at the 90^th^ percentile in the disability distribution) and shorter duration claims referred to VR specialists (reductions of 255 and 214 days at the 25^th^ and 50^th^ percentiles in the disability distribution, respectively).

**Conclusions:**

The WR program introduction was effective in reducing cumulative disability days paid for construction worker claims but the effects varied at different percentiles in the disability distribution, as well as by specialist referral. The findings highlight the benefits of better integrated RTW and VR services to injured workers in the construction sector.

Work-related injuries and illnesses are often more common in the construction sector than other industries. The construction sector has the highest injury claim counts and rates in most developed countries (1–3). Additionally, measures of return-to-work (RTW) and work disability outcomes are shown to be worse in construction than other sectors (4–6). RTW and vocational rehabilitation (VR) program interventions present opportunities to improve work disability outcomes for injured workers. While there is a substantial body of literature evaluating intervention effectiveness (7–12), studies have either been limited in methodology or not specifically focused on the construction sector.

RTW is best understood as an evolving process, comprising multiple phases, as opposed to a linear process with single independent events (13, 14). While in most cases RTW will encompass return to pre-injury employment with a pre-injury employer, it may require transitions to employment with a new occupation and new employer following VR interventions with educational training and work placement programs. Recent observational studies that have compared recipients and non-recipients of VR using propensity score matching (PSM) techniques have shown mixed results, including effectiveness (7, 15–17), ineffectiveness (18), or only effectiveness in particular segments of study populations (19, 20). While these studies provide valuable insight into the effectiveness of various VR programs and methodological advancement over previous research, there is an absence of evidence for program effectiveness in specific sectors (eg, construction) that face unique RTW challenges.

The construction sector is often associated with physically demanding work that is episodic, taking place across multiple worksites, and largely conducted by small firms. These characteristics, alongside others, can contribute to unique barriers to RTW. For example, small construction firms are reported to have greater difficulties in offering modified or alternate work for injured workers than large firms (21–23). Construction firms implementing disability management and RTW programs have reportedly found such programs to be costly, especially for providing new equipment, educating staff on programs and policies, and providing suitable duties (22). Only two studies have examined rehabilitation programs with construction workers. One focused on a three-week reconditioning program where just over half returned to their workplace and were still employed one year later (24). A second evaluated a workplace-based rehabilitation program to find a positive effect on RTW when workers were able to develop competent work behavior through progressive exposure to work, worksite assessments and work accommodations (25).

The purpose of this study was therefore to determine whether the introduction of a new integrated RTW and VR program offered by the Ontario Workplace Safety and Insurance Board (WSIB) was associated with reduced work disability duration in the construction sector.

## Methods

### Workers’ compensation and work reintegration

In Ontario, Canada, the WSIB is the organization responsible for providing wage-loss benefits, medical coverage and support to help workers get back to work after a work-related injury or illness. Ontario businesses fund the WSIB through premiums, and it provides no-fault collective liability insurance and access to industry-specific health and safety information for over five million people across more than 300 000 workplaces.

**Figure 1:**
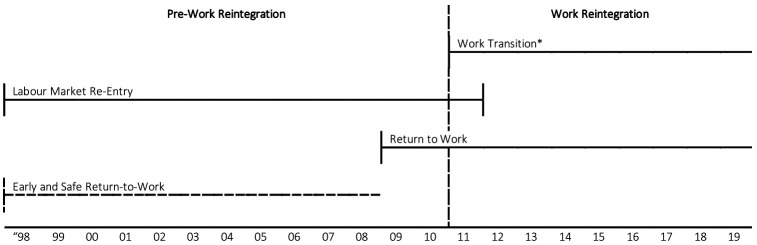
Timeline of the Workplace Safety and Insurance Board return-to-work and vocational rehabilitation programs and practices. *Work Transition services were introduced in December 2010 and replaced all Labour Market Re-Entry services by August 2011.

Between December 2010 and August 2011, the WSIB established Work Reintegration (WR), a program that replaced two separate RTW and VR programs and practices with a single integrated one (26). More specifically, the VR aspect of the WR program replaced the previous, externally managed, ’Labour Market Re-Entry’ services with internally managed ’Work Transition’ services. In terms of the RTW aspect of the WR program, the ’Early and Safe Return-to-Work’ practices that workplace parties were previously encouraged to follow were replaced with a new RTW service delivery model (see table 1 for key differences and figure 1 for timeline). Among several new features, the WR program introduced earlier RTW and VR interventions, penalties for worker and employer non-cooperation, program time limits, relocation assistance, and employment placement and retention support services (27). Examining the overall effect of the WR program introduction is therefore possible by comparing the work disability duration of claims that received VR services in the periods before and after the WR program introduction, with comparable claims that received RTW services or no services during the same periods.

**Table 1:** Summary of changes to the Workplace Safety and Insurance Board (WSIB) return-to-work (RTW) and vocational rehabilitation (VR)programs and practices

Pre-Work Reintegration	Work Reintegration
**Labour Market Re-Entry (LMR)**	**Work Transition**
WSIB gives RTW responsibility to workplace parties (worker and mainly original employer)	WSIB takes responsibility to ensure workers are re-integrated into decent, safe and sustainable work, either with original employer or in the open job market
WSIB has a monitoring and dispute resolution role	WSIB takes initiative in maintaining employment relationship
Case management and LMR services are outsourced	Internalized delivery of LMR case management; High standards of services delivered by WSIB and contracted providers
Limited academic options and lack of employment placement	Offers wide range of options for academic education/training; Introduces employment placement
**Early and safe return-to-work (ESRTW)**	**RTW**
Not integrated with LMR	Integrated LMR and RTW programs
Workplace parties are encouraged to follow ESRTW practices	

### Data

Employer, claim, payment, and program referral data were obtained from the WSIB for the years 2009 to 2017. Use of data for research purposes was governed by information sharing agreements between the WSIB and the research team, including data storage and access services provided by Population Data BC. Personal identifiers were removed from the data provided to the research team and replaced with anonymous claim identifiers. Ethical approval for the research project was obtained from the Behavior Research Ethics Board at the University of British Columbia (certificate number H13-00896). Data preparation and analyses were conducted using SAS 9.4 (SAS Institute Inc, Cary, NC, USA) and Stata 15.0 (StataCorp, College Station, TX, USA).

### Study cohort

The study population included workers with eligible claims for non-fatal, work-related injuries and musculoskeletal disorders (MSD) that occurred between the years 2009 and 2015, resulting in at least one day of compensated lost-time, in construction industry classifications. Benefit payments for all claims included a maximum follow-up time of two years, post-injury (including payments from years 2016 and 2017). Occupational disease (such as asthma and cancer) and mental health claims were excluded due to these claims typically having a longer time between exposure onset and claim registration. Workers had to be 15–80 years of age at the time of injury with complete information on analytical variables to be included in the cohort.

### Outcome measure

The outcome measure was cumulative disability days paid up to two years of the injury date. To ensure consistent follow-up, benefit payment dates up to 730 calendar days from the injury date were included. To account for different work schedules, adjustments were made to standardize cumulative disability days paid to a five-day workweek. Claims with more than 520 days (equivalent to two years based on a five-day workweek) were right-censored. This variable was chosen due its consistent measurement during the study period and availability for all claims.

### Treatment and control groups

During the study period, claims could be referred to RTW and VR specialists. In some cases, they could receive referrals multiple times and to different types of specialists offering different forms of services. Since the majority of claims that received VR referrals also received RTW referrals (80%), claims were separated into three distinct groups during the pre-program change period (2009–2010) and post-program change period (2011–2015): claims with no referrals (control group), claims with only RTW specialist referrals, and claims with VR specialist referrals that may or may not have also included RTW specialists (treatment groups). Due to data limitations, treatment and control groups and program periods were defined based on injury year and referral type, regardless of referral timing or services received.

### Propensity score matching

Workers that received RTW and VR specialist referrals were different from those not referred, with more severe injuries, and from particular occupations, industries, and firm sizes. To account for this selection bias, a matching method was used to construct comparable cohorts for claims from all three groups, prioritising the representation of VR specialist referral claims since the majority of program changes were specific to VR. Accordingly, the VR specialist referral group was matched with the other groups separately, dropping out claims with VR specialist referrals that did not share matching characteristics with those from the other groups. The proportions of the aggregate, unmatched cohort was approximately a ratio of 1:2:5 for referral to VR specialists, RTW specialists, and no referrals, respectively.

Considering the relative proportion of claims under each treatment, a nearest neighborhood matching approach was used (28), where one VR specialist referral claim was matched with a maximum of two nearest RTW specialist referral claims and five nearest no referral claims, respectively. Matching was conducted separately for the pre-program change period (2009–2010) and post-program change period (2011–2015) (see supplementary material, www.sjweh.fi/article/4006, for more details). After matching, three balanced cohorts were obtained for both periods, determined by propensity score estimates from a logit model with all observable characteristics contributing to treatment likelihood.

### Sociodemographic and work-related factors

Age at time of injury, year and quarter of injury date, injury type and severity proxy, occupation, pre-injury earnings, industry subsector, firm size and residential community size were used for matching. Injury data were coded using Canadian Standards Association Z795-03 coding for work injury and disease (29). Injury type was categorized into five groups similar to previous research (30, 31): non-MSD, back soft tissue injuries, other soft tissue injuries, limb fractures and other fractures. An injury severity proxy was created using predicted log cumulative disability days paid, based on a multi-jurisdictional multivariable regression model of injured construction workers from British Columbia, Alberta, Manitoba and Ontario, merged at the level of the claim using 2-digit part of body and 3-digit nature of injury codes. Occupation was coded to National Occupational Classification 2006 (32) and categorised into trade, transport and equipment operators and related occupations versus all other occupations. Quintiles were calculated for pre-injury gross annual earnings. Industry subsector was coded to the North American Industry Classification 2012 (33) and categorised into heavy and civil engineering construction versus construction of buildings/specialty trade contractors. Firm size was based on the number of full-time equivalents (FTE) and classified as small, medium, and large firms based on thresholds of <10, 10–99 and ≥100 FTE, respectively. The six-digit postal code of the worker at the time of injury was converted, using a Statistics Canada conversion file (34), to the 2011 Census community size code to distinguish between claims from large urban areas and those from smaller urban or rural areas.

### Difference-in-differences quantile regression

Cumulative disability days paid were compared for claims that received referrals to VR specialists, RTW specialists, and no referrals. A difference-in-differences (DiD) method was used and allowed for different treatment effects for the VR specialist group versus the RTW specialist only group (no referral claims represented the control group). The baseline estimation model was:


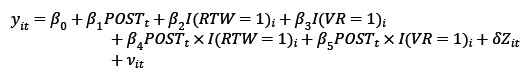


where *y_it_* was the cumulative disability days paid for claim *i* at time *t*, *POST_t_* was an indicator equal to 1 if the claim was observed after 2010, *I(RTW=1)_i_* and *I(VR=1)_i_* were indicators for being in the RTW specialist group, or VR specialist group respectively. *Z_it_* is a vector of claim-level observable characteristics as described above. *ν_it_* are random error terms. The parameters of interest were *β_4_* and *β_5_*, which identified the effects of the RTW and VR referrals on cumulative disability days paid. The inverse propensity weights imposed on this model were calculated from the matching.

The DiD method relies on the parallel trends assumption that control and treatment groups have similar pre-trends and would have continued on the same paths had the intervention not taken place. To test this assumption, the trends in pre-program change period cumulative disability days paid were compared for each group and presented a comparable downward trend for all three groups. Since cumulative disability days paid is a highly skewed measure, with a large proportion of claims receiving few disability days and a small proportion continuing to accumulate disability days during the two years following injury, linear regression was used to model differences at the mean whereas quantile regression was used to model differences at the 25^th^, 50^th^, 75^th^ and 90^th^ percentiles of the distribution. This approach follows previous work disability and RTW studies that have relied on similar skewed outcome data (30, 31, 35). Each regression model is adjusted for the same variables used in the propensity score matching, with the exception of the injury severity proxy.

## Results

Descriptive statistics of the claim cohorts show how the control and treatment groups across the entire study period were different (table 2). Overall, in the unmatched cohorts, compared to claims with RTW specialist referrals or no referrals, claims with VR specialist referrals had a larger proportion of fracture-related injuries, higher pre-injury earnings, worked for smaller sized firms, were of older age at the time on injury, were paid more cumulative disability days paid, and had higher predicted disability cumulative disability days paid based on their injury characteristics. Following matching, there was greater balance on the preceding characteristics.

**Table 2a:** Descriptive statistics of work-related injury and musculoskeletal claims in the construction sector for the control group and treatment groups before and after matching. [No referral=no referral to return-to-work or vocational rehabilitation specialists. RTW referral=return-to-work specialist referral; VR referral=vocational rehabilitation specialist referral; MSD=musculoskeletal disorder.]

	Unmatched (N=26 532)			Matched (N=12 909)		
	
	No referral (N=17 138)	RTW referral (N=5916)	VR referral (N=3478)	No referral (N=4345)	RTW referral (N=5086)	VR referral (N=3478)
	
	%	%	%	%	%	%
Sex						
Male	97.5	97.2	97.9	98.1	97.9	97.9
Female	2.5	2.8	2.1	1.9	2.1	2.1
Injury type						
Non-MSD	39.0	20.7	18.4	17.0	18.7	18.4
Back soft-tissue	20.8	21.0	13.1	13.2	13.3	13.1
Other soft tissue	28.6	30.5	33.4	36.8	33.2	33.4
Limb fracture	1.5	5.0	7.0	6.3	6.8	7.0
Other fracture	10.1	22.8	28.1	26.7	28.0	28.1
Injury year						
2009	16.5	10.2	14.2	14.6	14.3	14.2
2010	16.2	9.7	10.7	10.3	10.6	10.7
2011	14.0	15.5	10.6	10.4	11.1	10.6
2012	12.9	17.3	12.1	11.8	11.7	12.1
2013	12.9	18.4	18.0	17.8	18.3	18.0
2014	13.6	15.4	17.9	17.7	17.8	17.9
2015	13.9	13.5	16.5	17.4	16.2	16.5
Injury quarter						
1^st^	21.8	21.8	20.9	22.1	20.8	20.9
2^nd^	24.6	23.5	23.8	22.9	24.3	23.8
3^rd^	29.2	29.2	29.4	29.3	29.1	29.4
4^th^	24.4	25.5	25.8	25.7	25.9	25.8
Occupation						
Trades, transport, equipment operators & related occupations	89.2	90.8	91.2	90.8	91.3	91.2
Other	10.8	9.2	8.8	9.2	8.7	8.8
Earnings quintile						
1^st^	20.0	20.2	18.9	20.4	19.0	18.9
2^nd^	20.3	19.3	16.7	15.5	16.7	16.7
3^rd^	20.1	19.2	19.8	19.3	20.1	19.8
4^th^	20.1	21.3	21.9	22.1	21.3	21.9
5^th^	19.5	20.0	22.7	22.7	22.8	22.7
Industry						
Construction of buildings / specialty trade contractors	89.9	88.6	88.3	87.4	88.4	88.3
Heavy & civil engineering construction.	10.1	11.4	11.7	12.6	11.6	11.7
Firm size ^a^						
<10	46.5	51.7	56.8	57.0	56.7	56.8
10–99	38.2	36.4	31.6	30.8	31.3	31.6
≥100	15.3	11.8	11.6	12.2	12.1	11.6
Community size						
≥1 500 000	33.5	39.1	33.3	32.2	32.5	33.3
500 000–1 499 999	15.8	12.6	14.0	14.7	14.1	14.0
100 000–499 999	24.9	24.6	26.6	26.6	26.7	26.6
10 000–99 999	9.3	9.0	11.0	11.2	11.1	11.0
<10 000	16.5	14.7	15.0	15.3	15.5	15.0

a Full-time equivalents (FTE)

**Table 2b:** Descriptive statistics of work-related injury and musculoskeletal claims in the construction sector continued.

	Unmatched (N=26 532)						Matched (N=12 909)					
	
	No referral (N=17 138)		RTW referral (N=5916)		VR referral (N=3478)		No referral (N=4345)		RTW referral (N=5086)		VR referral (N=3478)	
					
	Mean	SD	Mean	SD	Mean	SD	Mean	SD	Mean	SD	Mean	SD
Age	36.8	12.4	39.6	12.2	42.4	11.9	43.2	12.9	42.4	12.3	42.4	11.9
Predicted disability days paid	18.2	17.9	30.2	25.1	39.1	30.8	37.9	31.9	38.8	30.7	39.1	30.8
Disability days paid	17.2	45.8	78.2	80.8	333.5	170.9	31.7	74.1	87.8	91.9	333.5	170.9
Pre-accident annual earnings	50 154.7	23 660.4	50 672.8	24 118.1	53 676.2	27 554.7	52 078.6	25 436.9	52 378.0	24 924.7	53 686.2	27 554.7
FTE^a^	75.3	221.4	60.1	194.8	52.6	154.1	64.8	219.4	59.2	189.7	52.6	154.1

a Full-time equivalents (FTE)

Table 3 summarizes the pre-program change differences in cumulative disability days paid between the three groups, as well as their post-program change differences, and the DiD (pre-post differences for the treatment groups relative to pre-post differences for the control group) at the mean, 25^th^, 50^th^, 75^th^ and 90^th^ percentiles of the disability distribution, after matching and adjusting for individual-level characteristics. Claims from the three groups had fundamentally different cumulative disability days paid from each other, even after matching on observable characteristics. Claims referred to RTW and VR specialists had, on average, 108.5 [95% confidence interval (CI) 96.5–120.5] and 390.4 (95% CI 380.0–400.8) more paid disability days, respectively, than claims with no referrals during the pre-program change period. These differences were relatively consistent across the disability distribution. Following the program change, claims from all three groups were paid less cumulative disability days. Claims referred to RTW and VR specialists experienced larger reductions in their cumulative disability days paid than claims with no referrals but the effects differed by percentiles in the disability distribution. RTW referral claims with longer durations experienced the largest reduction in cumulative disability days paid whereas VR referral claims with shorter durations experienced the largest reductions. For example, claims referred to RTW specialists had 204.36 (95% CI -253.55– -155.17) fewer paid disability days at the 90^th^ percentile whereas claims referred to VR specialists had 255.4 (95% CI 276.6–234.2) fewer paid disability days at the 25^th^ percentile and 213.9 (95% CI 228.0–199.80) at the 50^th^ percentile.

**Table 3:** Multivariable difference-in-differences linear and quantile regression models estimating the effectiveness of the Work Reintegration program on cumulative disability days paid during two-years, post-injury, among matched cohorts of work-related injury and musculoskeletal disorder claims in the Ontario construction sector, 2009-2015 (N=12 909 claims). Models adjusted for age, sex, injury type, injury year, injury quarter, occupation, pre-injury earnings, industry, firm size, and community size. [CI=confidence interval; No referral=no referral to return-to-work (RTW) or vocational rehabilitation (VR) specialists; RTW referral=RTW specialist referral; VR referral=VR specialist referral.]

Program referral	All R^2^=0.63		25^th^ percentile Pseudo R^2^=0.30		50^th^ percentile Pseudo R^2^=0.45		75^th^ percentile Pseudo R^2^=0.59		90^th^ percentile Pseudo R^2^=0.53	
				
	Coef	95% CI	Coef.	95% CI	Coef.	95% CI	Coef.	95% CI	Coef.	95% CI
**Pre-program change period**										
Constant	49.20	34.78–63.62	7.78	5.57– 9.99	15.45	12.20– 18.71	42.15	34.45– 49.85	109.19	88.55– 129.84
No referral		Reference		Reference		Reference		Reference		Reference
RTW referral	108.45	96.46– 120.45	57.95	51.86– 64.04	94.49	87.60– 101.38	136.78	117.93– 155.62	274.17	225.57– 322.77
VR	390.40	380.04– 400.76	388.27	368.79– 407.75	484.78	480.85– 488.72	473.91	468.30– 479.52	422.68	402.70– 442.65
**Post-program change period**									
Post (Ref: No referral	-47.09	-57.10– -37.08	-6.28	-8.15– -4.41	-10.97	-13.36– -8.58	-29.15	-34.93– -23.37	-69.75	-90.61– -48.88
Post # RTW referral	-70.30	-83.23– -57.37	-33.54	-39.80– -27.28	-54.34	-61.57– -47.10	-78.46	-97.52– -59.41	-204.36	-253.55– -155.17
Post # VR referral	-118.66	-131.42– -105.90	-249.16	-270.33– -227.98	-202.92	-216.98– -188.85	-21.99	-30.21– -13.77	31.87	11.26– 52.48
**Pre-Post differences**										
No referral	-47.09	-57.1– -37.08	-6.28	-8.15– -4.41	-10.97	-13.36– -8.58	-29.15	-34.93– -23.37	-69.75	-90.61– -48.88
RTW referral	-117.39	-130.47– -104.31	-39.82	-46.27– -33.37	-65.31	-72.65– -57.97	-107.61	-126.05– -89.18	-274.11	-319.02– -229.19
VR referral	-165.75	-177.82– -153.69	-255.44	-276.63– -234.24	-213.89	-227.96– -199.82	-51.14	-57.34– -44.94	-37.88	-41.41– -34.35

**Figure 2:**
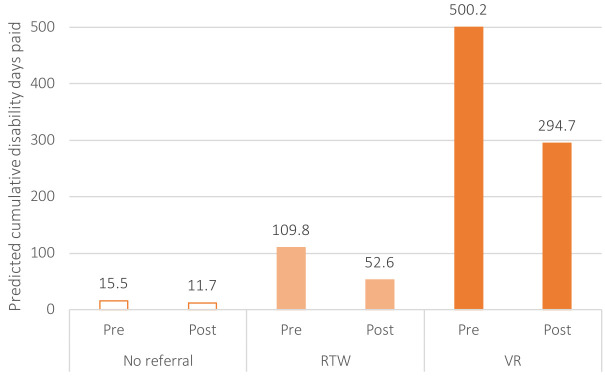
Predicted cumulative disability days for work-related injury and musculoskeletal disorder claims in the Ontario construction sector, by pre and post-program change period and program referral, based on a multivariable difference-in-differences quantile regression model at the 50^th^ percentile of the disability distribution using matched cohorts, 2009–2015.

Using the estimates from the multivariable quantile regression at the 50^th^ percentile of the disability distribution, the predicted disability days paid were obtained for the pre- and post-intervention periods for all three groups (figure 2). The introduction of the WR program resulted in a decline of cumulative paid disability days from 15.5 to 11.7 for claims with no referral, and from 109.8 to 52.6 and 500.2 to 294.7 for claims referred to RTW and VR specialists, respectively. In other words, claims at the median of the distribution would have experienced a drop of approximately 200, 50 and <5 days if they were referred to VR specialists, RTW specialists or no specialists, respectively.

## Discussion

The purpose of this study was to determine whether the introduction of the WSIB WR program was associated with reduced work disability duration in the construction sector in Ontario. Using a DiD quantile regression approach with matched cohorts, the findings suggest that the program reduced cumulative disability days paid for all claims and particularly for longer duration claims (90^th^ percentile of the disability distribution) referred to RTW specialists and shorter duration claims (25^th^ percentile of the disability distribution) referred to VR specialists.

Unlike VR programs in most other studies, the WR program is an integrated RTW and VR program offered to individuals with work disability due to work-related injury and illness and within the context of a no-fault workers’ compensation system. Furthermore, unlike most existing studies, this study examined the effectiveness of the program within the context of the construction sector and with regards to changes in cumulative disability days paid. Some studies have used measures related to insurance benefit receipt, such as those based on the probability of individuals no longer receiving disability insurance benefits and shown evidence of effectiveness (17, 36). For example, a study of social security disability insurance beneficiaries in the United States found that individuals receiving services from state VR agencies had a higher rate of completing a trial work period and achieving suspension or termination from the disability insurance program due to work than their matched counterparts (17). A Canadian study evaluating the effectiveness of a VR program provided to Canadian pension plan disability insurance beneficiaries found no effect of exiting disability benefits or on obtaining employment among men compared to their matched counterparts. However, the study did find an increase in obtaining substantial gainful employment among women compared to their matched counterparts (36). In contrast to these studies, the present study evaluated the effectiveness of an integrated RTW and VR program available to much broader population and found a robust effect in reducing the number of disability days paid to injured workers in the construction sector.

Understanding why the WR program was more effective for long-duration claims referred to RTW specialists and short-duration claims referred to VR specialists is complex. It is possible that injured workers with these types of claims were more receptive to program services whereas those with long duration claims referred to VR specialists were more likely to experience persistent and significant disability as they may have had more severe injuries, which made ever achieving RTW unlikely. The timing and length of interventions can be key factors in the effectiveness of VR programs. A study evaluating a VR program for individuals with MSD and mental-related work disability within the Finnish earnings-related pension scheme found that among those with shorter rehabilitation (≤10 months), the largest gains in work participation were observed in the year after rehabilitation, after which it decreased. In contrast, among those with longer rehabilitation (>10 months), increasing gains were observed with each follow-up year. While the WR program has thresholds in which specific activities should take place – such as following an injury having (i) a RTW specialist meeting in ≤12 weeks, (ii) an initial meeting with a case manager and work transition specialist within 6–9 months, or (iii) a work transition plan approved in less than one year (27) – there may still be variation in the timing of these events and their affiliated support services that can further explain differences in overall program effectiveness.

The findings suggest that despite there being evidence of program effectiveness in the construction sector, there may still be challenges for injured construction workers returning to work, particularly among those with long disability duration. There are barriers to RTW that are unique to the construction sector that may help explain this. As highlighted in a scoping review (Sharpe et al, unpublished manuscript), these barriers often relate to the offer of work accommodations, such as modified work or alternative duties that are more more challenging given the physical nature of construction work (21–23). Limited transferable skills may act as a barrier to work accommodations and VR, particularly since the sector includes specialized occupations that may limit opportunity to offer alternative work outside an injured worker’s skillset. Organizational factors, including firm size, also shape RTW. For example, construction firms are typically small and small firms face greater difficulties than large firms in accommodating injured workers (21–23). Other notable barriers the authors highlight include a lack of understanding of the nature of construction work among healthcare providers, leading to difficulties in identifying injured worker’s restrictions and capabilities (22), and how construction workers may accept injuries as part of work in the industry and work through pain to remain in the workplace due to normative expectations of masculinity and stoicism in the sector (37).

The findings suggest that efforts to better integrate RTW and VR services through the WR program have resulted in improvements (reductions) in cumulative disability days paid to injured construction workers. However, it is important to acknowledge that cessation of disability benefits is not necessarily a measure of successful RTW. Workers may follow complex RTW trajectories, comprising multiple phases (13, 14). Furthermore, it should be acknowledged that over time the WSIB and many other disability insurers have changed their focus to include workers’ abilities to work or be deemed “employable”. Consequently, while a worker may no longer be claiming disability benefits and is deemed “employable”, they may still have unresolved medical problems, giving them a disadvantage in the labor market (38). Complementing the administrative data-based approach in this study with more qualitative approaches that take into account the perspective of the workers’ experience would further understandings of the extent to which the WR program has improved RTW of injured construction workers and where improvements to the program can still be made.

### Strengths and limitations

This study is the first to evaluate the effectiveness of the WR program. A unique contribution of this study was the use of quantile regression to examine differences in the effectiveness of an integrated RTW and VR program by varying levels of disability duration. This contrasts with the majority of evaluations that have focused on VR intervention effects on employment probabilities (7, 15, 18, 20). By using this approach, the study shows how the WR program was associated with a reduction in the cumulative disability days paid of longer duration claims referred to RTW specialists and shorter duration claims referred to VR specialists. This finding is important from a policy and practice perspective as many injured workers benefitted from the program in terms of reducing disability duration, whereas those with more severe injuries or disability may still experience barriers to RTW, indicated by smaller reductions in disability duration among longer duration claims referred to VR specialists. The combination of a DiD with PSM matching algorithm provides a robust study design that other observational studies of VR programs have also used (7, 16).

There are however limitations to how the program periods were measured and the treatment and control groups within them. Due to the program periods being based on injury year, as well as the phase-out period of claims under the old program, there is a degree of overlap between claims in both periods and lag in program effect measurement. However, it is more likely that the overall effectiveness of the WR program was underestimated as a result of this as there was likely a larger number of claims from the old program, receiving fewer, externally managed services, grouped with those of the new program, reducing the treatment effect. Since the control and treatment groups were based on specialist referral, as opposed to when the referrals were made or what services were received and when, this study was unable to determine whether the reduction in disability days was due to earlier referrals or other program changes (eg, increased services). Nonetheless, the measurements used serve as proxies for estimating overall program effectiveness.

It should be noted that on 1 January 2013, the WSIB expanded compulsory workers’ compensation coverage to independent operators, sole proprietors, partners in a partnership, and executive officers of corporations carrying out business in the construction sector (39). However, given that these groups are less likely to file claims than larger construction firms, financially incentivized to not claim disability benefits in the long-term (ie, experience rating), and the fact that the matching approach included matching on firm size, any potential bias in the results introduced by this change is thought to be minimal. There is the possibility of unobserved heterogeneity between the treatment and control groups that may bias the results. This is a common limitation of relying solely on administrative data. Lastly, the outcome variable only provided a proxy for RTW as claims no longer receiving payments for cumulative disability days do not necessarily result in RTW and could instead be in receipt of disability pension, other employer-based renumeration (sick pay), or no renumeration. Since the outcome variable was cumulative, claims with the same disability days paid may have accumulated the days over different calendar periods. It is also possible that differences observed over time for long duration claims (eg, 90^th^ percentile of the disability distribution) may have been underestimated due to the censoring of data at two-years post-injury. However, this was a necessary methodological decision in order to create comparable cohorts over time.

### Concluding remarks

The findings suggest that the WSIB WR program introduction was effective in reducing cumulative disability days paid for injury claims among construction workers referred to RTW and VR specialists. While the effects varied at difference percentiles in the disability distribution, as well as by type of program referral, further research could examine the type and timing of services received to understand what specific changes in the WR program contribute to the overall findings.

## Supplementary material

Supplementary material
