# Supplementary material for: Evaluating effectiveness of an integrated return-to-work and vocational rehabilitation program on work disability duration in the construction sector
Source: Scand J Work Environ Health. 2022 Mar 31;48(3):229–38. doi: 10.5271/sjweh.4006 (PMC9523466; doi:10.5271/sjweh.4006)
Supplement: Supplementary material [file SJWEH-48-229-S001.pdf]

Evaluating effectiveness of an integrated return-to-work and vocational rehabilitation program on work disability duration in the construction sector[1]

by Robert A Macpherson, PhD,[2] Ailin He, PhD, Benjamin C Amick III, PhD, Mieke Koohoorn, PhD, Christopher B McLeod, PhD

1. *Supplementary material*
2. *Correspondence to: Dr Robert A Macpherson, School of Population and Public Health, University of British Columbia, 2206 East Mall, Vancouver, BC Canada, V6T 1Z3. [E-mail: robert.macpherson@ubc.ca]*

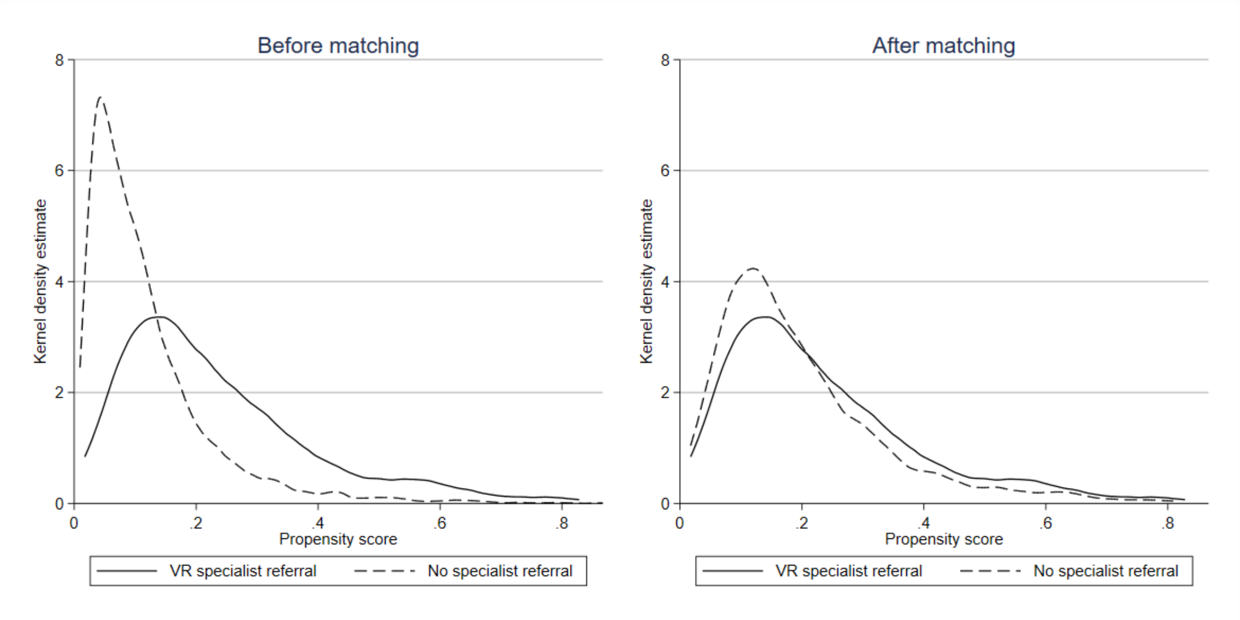

Supplementary Figure 1. Propensity score distributions of treatment and control groups before and after matching during the pre-program change period.

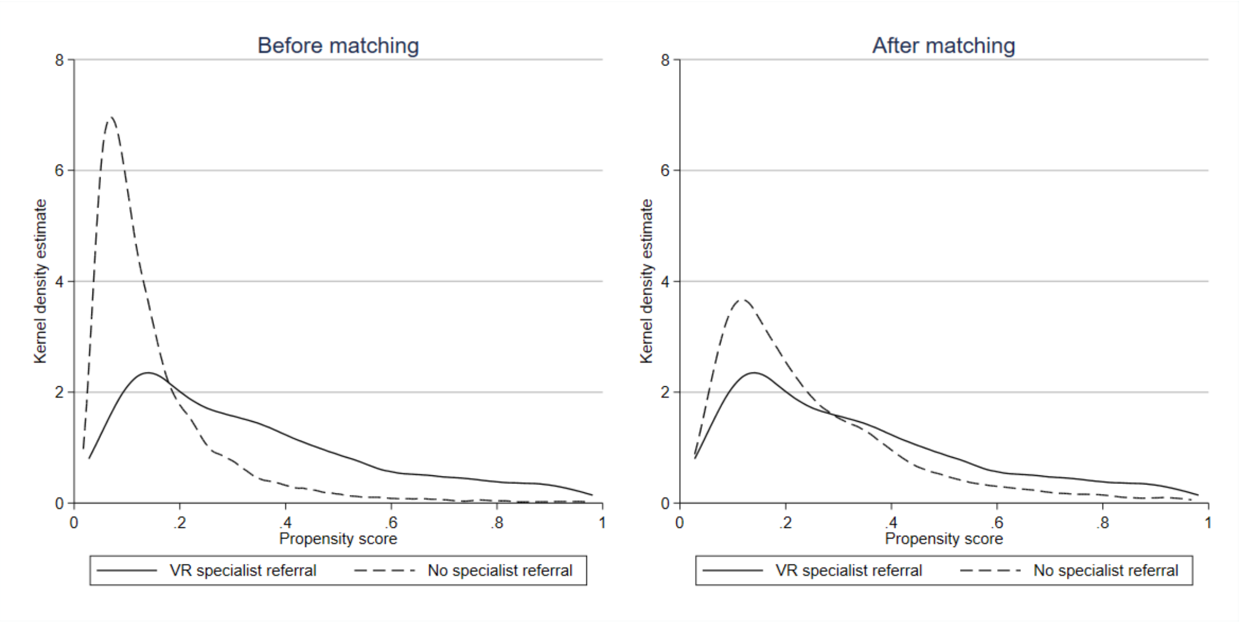

Supplementary Figure 2. Propensity score distributions of treatment and control groups before and after matching during the post-program change period.

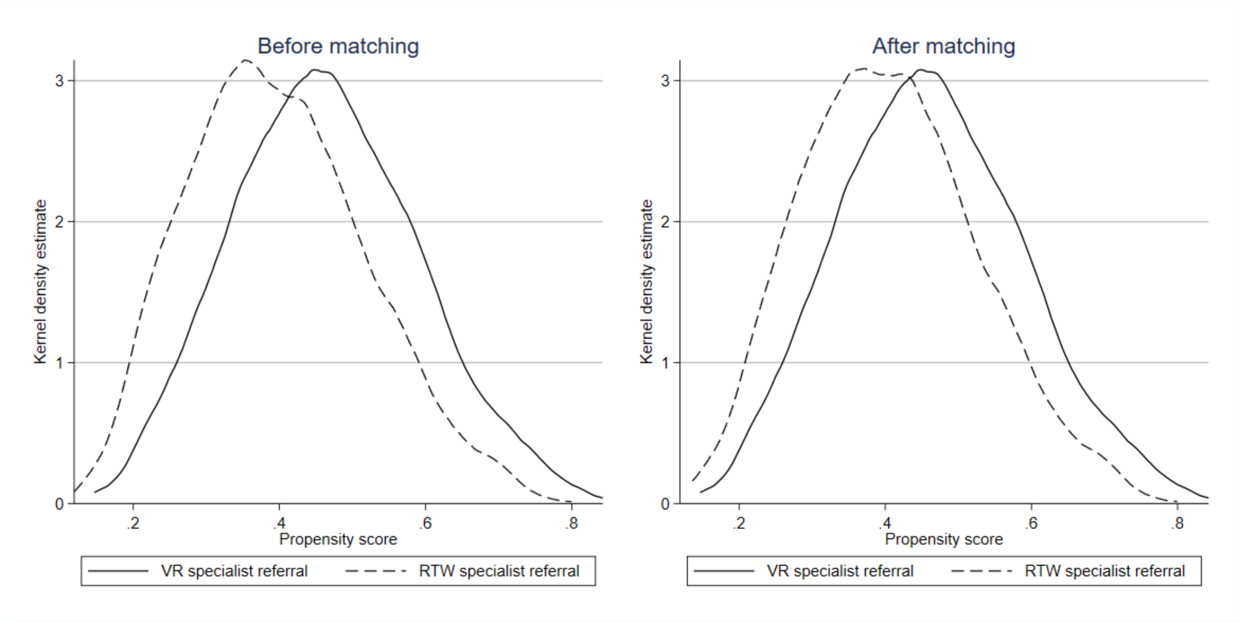

Supplementary Figure 3. Propensity score distributions of treatment and control groups before and after matching during the pre-program change period.

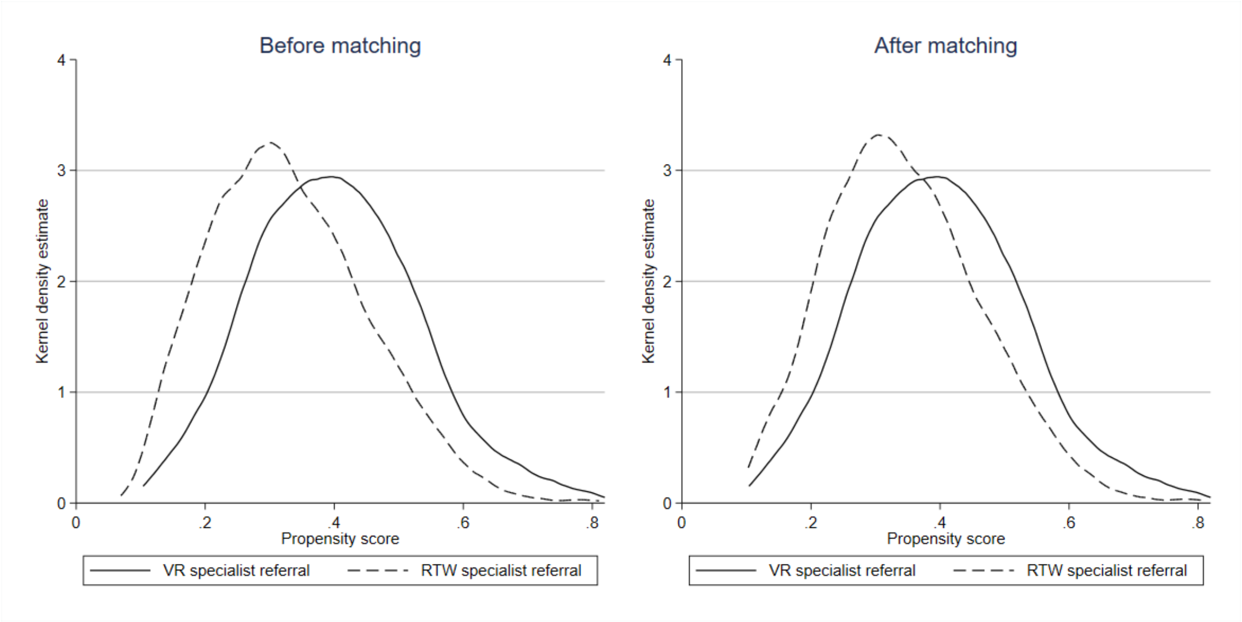

Supplementary Figure 4. Propensity score distributions of treatment and control groups before and after matching during the post-program change period.
